# DNA methylation ambiguity in the *Fibrillin-1* (*FBN1*) CpG island shore possibly involved in Marfan syndrome

**DOI:** 10.1038/s41598-020-62127-3

**Published:** 2020-03-24

**Authors:** Yoshikazu Arai, Kazuhiro Umeyama, Natsumi Okazaki, Kazuaki Nakano, Koichiro Nishino, Hiroshi Nagashima, Jun Ohgane

**Affiliations:** 1 Laboratory of Veterinary Biochemistry and Molecular Biology, Faculty of Agriculture, University of Miyazaki, Miyazaki, 889-2192 Japan; 2 Laboratory of Developmental Engineering, Department of Life Sciences, School of Agriculture, Meiji University, Kawasaki, 214-8571 Japan; 3 Meiji University International Institute for Bio-Resource Research (MUIIBR), Kawasaki, 214-8571 Japan; 4 Laboratory of Genomic Function Engineering, Department of Life Sciences, School of Agriculture, Meiji University, Kawasaki, 214-8571 Japan; 5 Center for Animal Disease Control, University of Miyazaki, Miyazaki, 889-2192 Japan

**Keywords:** DNA sequencing, DNA methylation

## Abstract

*Fibrillin-1* (*FBN1*) is responsible for haploinsufficient and autosomal dominant Marfan syndrome. Even in the same Marfan pedigree, penetrance and expressivity in heterozygous individuals can differ and result in variable disease onset and severity. Thus, other factors in addition to mutations in *FBN1* are likely to contribute to the disease. In this study, we examined the regulation of *FBN1* in porcine Marfan syndrome model, focusing on DNA methylation patterns distinguishable as wild-type (WT) and *FBN1* null (KO) alleles in heterozygous cells. Most importantly, the ratio of the transcriptionally active hypomethylated WT allele was altered during cellular passage and highly correlated with *FBN1* mRNA level compared with that in the KO allele. Transcribed *FBN1* RNA from the KO allele was abolished after splicing coupled with translational initiation, suggesting that the functional *FBN1* mRNA levels were affected by DNA methylation of the WT allele.

## Introduction

*Fibrillin-1* (*FBN1*) encodes the microfibril protein, fibrillin, which is expressed in various connective tissues and is a responsible gene for Marfan syndrome^1,2^. Marfan syndrome is caused by the inheritance of one mutated null allele and classified as autosomal dominant^3^. However, Marfan patients exhibit phenotypic variations even in the same pedigree with an identical mutated null allele^1,4^. In addition, patients with low levels of the *FBN1* expression suffer an increased risk of ectopia lentis, pectus excavatum, and aorta abnormalities^5^. Therefore, the expression level of *FBN1*, which is transcribed and translated into a functional fibrillin protein, is associated with disease onset and the severity of Marfan syndrome.

One cause of Marfan syndrome is haploinsufficiency, a condition with one mutated null allele and one normal allele. Haploinsufficiency is induced by a decrease in the amount of functional protein to less than half that produced under diploid expression^6^. Our Marfan model pigs^7^ exhibit haploinsufficiency with phenotypic variations in disease onset, mimicking the condition in humans, wherein differences in disease phenotypes or affected tissues have been reported even among patients from the same pedigree. In contrast, inheritance of two null mutant alleles is sufficient to cause autosomal recessive diseases. Thus, unlike autosomal recessive diseases, Marfan syndrome caused by autosomal dominant haploinsufficiency are unlikely explained completely by genetic mutations, and the molecular mechanism(s) that cause such phenotypic variation must be elucidated.

DNA methylation in transcriptional regulatory regions is crucial for tissue/cell type-specific gene expression^8–10^ and plays an important role in cellular differentiation and mammalian development^11–13^. Sodium bisulfite sequencing is a conventional method for DNA methylation analysis and identifies DNA methylation patterns in each sequenced fragment that can be recognized as one allele (cell) in a tissue or cell population^14,15^. Based on this concept, we previously focused on transcriptionally active reduced methylation allele (RM allele), which was described as DNA hypomethylated alleles (Hypo-alleles) in our previous study^14,15^ that can be recognized by the high ratio of unmethylated CpGs in each sequenced fragment (allele), and the RM allele ratio of a non-imprinted autosomal gene can be used to estimate the proportion of the cell type expressing that gene in the tissue or cell population^14,15^. Therefore, the RM allele model can be applied to detect transcriptional regulation of each allele in diploid cells in certain cell populations.

In human Marfan patients, an identical *FBN1* null mutant allele is inherited in a pedigree and can present different disease onset and severity among individuals in the same family. In humans, genetic sequences differ among individuals in the same pedigree, making it impossible to eliminate influences of genetic differences other than that of the gene responsible for the disease on the variations of disease phenotypes in affected individuals. This hinders the ability to elucidate the cause of phenotypic variations of genetic diseases. At present, differences in nucleotide sequences of genetic modifiers are thought to be responsible for phenotypic variations such as varied disease onset and/or severity in genetic diseases. In contrast, we produced *FBN1* heterozygous knockout (het KO) cloned pigs using genome editing and somatic cell nuclear transfer as an animal model for human Marfan syndrome^7^. Previous studies on Marfan patients have reported two types of *FBN1* genetic mutations, haploinsufficiency and dominant-negative^16^. Although both types of *FBN1* mutations are important for understanding the pathogenic mechanisms of Marfan syndrome, disease onset of our *FBN1* KO cloned pig model was caused by haploinsufficiency. This was confirmed by the absence of mutant mRNA with a premature stop codon^7^. These Marfan model pigs exhibit a broad spectrum of abnormal phenotypes that are also found in humans^7^. The *FBN1* het KO cloned pigs each have a syngeneic background because of their production by somatic cell nuclear transfer; however, these Marfan model cloned pigs exhibit a wide range of disease phenotypes including non-penetrance. Thus, the disease onset and severity of the Marfan model cloned pigs most likely result solely from a genetic mutation by a 1-bp deletion within the *FBN1* coding region that causes a frameshift and creates a premature stop codon^7^ and not from genomic sequence differences outside *FBN1*.

In our previous study, we reported that the porcine *FBN1* is regulated by DNA methylation, and *FBN1* mRNA levels are associated with RM allele ratios of an *FBN1* upstream region of the CpG island shore^17^. We also found that the nucleotide sequence of the CpG island shore of porcine *FBN1* is more highly homologous to human *FBN1*, including the number of methylatable CpG dinucleotides than the mouse *Fbn1*. These results together with the results of *FBN1* het KO cloned pigs suggest the possibility that *FBN1* expression is regulated by variable changes in epigenetic modification in Marfan patients. To further investigate the epigenetic regulation of *FBN1* in cells with one mutated *FBN1*-null allele, which is similar to human Marfan syndrome, an experimental tool for allele-distinguished DNA methylation analysis was designed by producing *FBN1* het KO pigs in which the *FBN1* CpG island shores of the WT and KO alleles have a single nucleotide polymorphism (SNP). Here, we analyzed primary fibroblast cells and adult tissues derived from *FBN1* het KO pigs with the allele-distinguishable SNP in the CpG island shore and focused on RM allele ratios within the *FBN1* CpG island shore, especially in the WT allele.

## Results

### Establishment of primary fibroblast cell lines derived from *FBN1* het KO pigs with a SNP that differentiates normal (WT) and *FBN1* null (KO) alleles

In an *FBN1* homozygous KO pig produced by crossing *FBN1* het KO pigs^7^, no *FBN1* mRNA was detected by RT-PCR and real-time RT-PCR (Supplementary Fig. 1a,b). However, an *FBN1* preRNA that contained exons and introns before splicing was clearly detected in the *FBN1* homozygous KO cells and WT cells (Supplementary Fig. 1a), indicating that *FBN1* is transcribed from both normal (WT) and *FBN1*-mutated null (KO) alleles, while the spliced and mature *FBN1* mRNA detected by RT-PCR analysis was the transcript only from the WT allele. Thus, RNA from the KO allele apparently existed before splicing, whereas spliced mature KO mRNA became degraded by a nonsense-mediated RNA decay mechanism that recognizes the premature stop codon created by the 1-bp deletion within the *FBN1* coding region of the KO allele. This also indicates that, at least in our Marfan model pigs, dominant negative effects of the KO allele are not likely to be responsible for disease onset and severity due to absence of mature mRNA. In addition, treatment of the *FBN1* het KO fibroblast cells (KO-239-2 and KO-239-4) with 5-aza-2′-deoxycytidine (5-aza-dC), an inhibitor of DNA methyltransferase, caused a significant increase in the amount of *FBN1* mRNA (Supplementary Fig. 2a). These results suggest that transcription of *FBN1* mRNA from the WT allele is controlled by DNA methylation. Furthermore, our promoter assay combined with *in vitro* DNA methylation of the *FBN1* CpG island shore revealed that promoter activity was clearly reduced by DNA methylation of the *FBN1* CpG island shore (Supplementary Fig. 2b). These results indicate that *FBN1* is regulated, at least in part, by DNA methylation of its CpG island shore.

**Figure 1:**
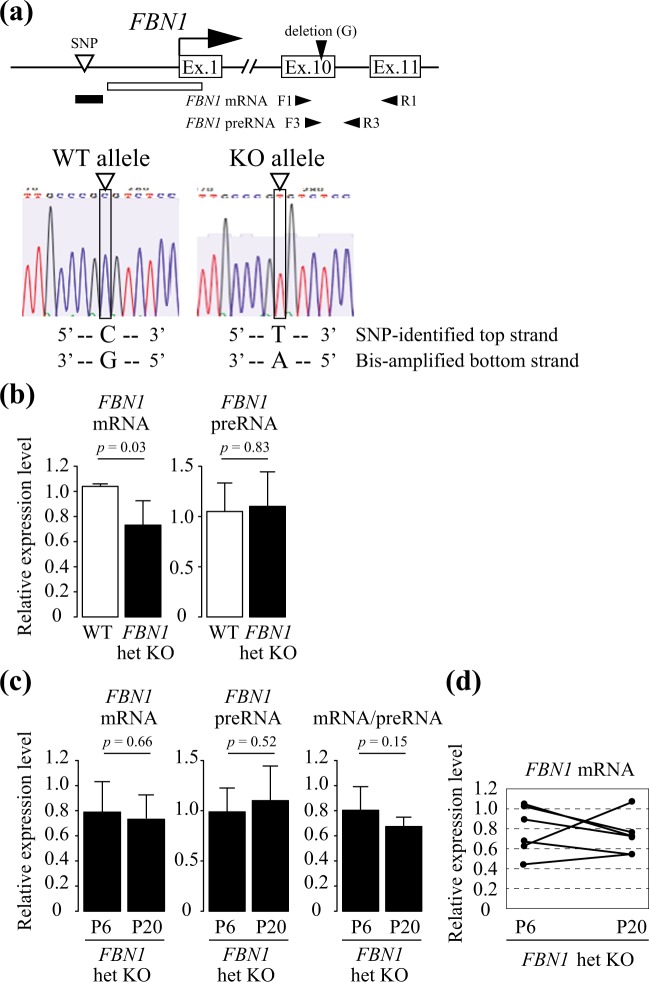
*FBN1* mRNA and preRNA levels in porcine *FBN1* het KO fibroblast cells depending on the normal (WT) and *FBN1*-mutated null (KO) alleles. (**a**) The upper panel shows a schematic of SNP and RT-PCR primer positions in the porcine *FBN1* locus. The RT-PCR primers were designed to amplify regions including the 1-bp deletion site (guanine, G) in the *FBN1* exon 10. *FBN1* mRNA and preRNA were amplified with the specific primer sets (F1 and R1) and (F3 and R3), respectively. White and black squares indicate the *FBN1* CpG island and the CpG island shore, respectively. The lower panel shows sequencing data of the SNP position in the *FBN1* CpG island shore. Open triangles indicate the position of the SNP-identified top strand in the *FBN1* het KO pigs (C: WT allele, T: KO allele). The same position of the SNP in the complementary strand is G in the WT allele and A in the KO allele, and was amplified by sodium bisulfite PCR. (**b**) Expression levels of the *FBN1* mRNA and preRNA in wild-type fibroblast cells (n = 3) and *FBN1* het KO fibroblast cells (n = 6) were evaluated by RT-PCR. (**c**) Expression levels of the *FBN1* mRNA, preRNA, and ratio of mRNA to preRNA (mRNA/preRNA) in *FBN1* het KO fibroblast cells (n = 6) collected at passages 6 and 20 (P6 and P20) were measured by RT-PCR. (**d**) Changes of *FBN1* mRNA levels from P6 to P20 in each *FBN1* het KO fibroblast cell line (n = 6). These data are based on the *FBN1* mRNA data in (**c**). The relative expression levels of *FBN1* mRNA and preRNA in (**b**,**c**) were normalized to *GAPDH* expression and shown as mean ± SD (n = 3). Statistical comparisons of the expression levels were performed using the Student’s *t*-test, and statistical significance was set as *p* < 0.05.

In order to investigate the regulation of *FBN1* mRNA level in porcine *FBN1* het KO cells, we attempted to analyze DNA methylation status of the *FBN1* CpG island shores of WT and KO alleles, independently. As a tool to perform allele-distinguished DNA methylation analysis of the *FBN1* CpG island shore, we first produced *FBN1* het KO pigs that contained an allele-distinguishable single nucleotide polymorphism (SNP) within the *FBN1* CpG island shore. Comparison of the genomic sequences of the *FBN1* CpG island shore in WT and *FBN1* het KO pigs could successfully locate a SNP that is a cytosine in both alleles of WT pigs and a thymine in both alleles of the *FBN1* het KO pigs. We produced F1 *FBN1* het KO pigs that had both cytosine and thymine SNPs that corresponded to WT and KO alleles, respectively (Fig. 1a). Using neonatal tail-cartilage from SNP-containing *FBN1* het KO pigs, we established primary fibroblast cell lines from six F1 het KO pigs (KO-239-2, KO-239-3, KO-239-4, KO-239-5, KO-241-2, and KO-241-5) as well as fibroblast cell lines from two F1 WT littermates (WT-239-1 and WT-241-1). Analysis of *FBN1* expression in these fibroblast cell lines by RT-PCR revealed that the amount of the *FBN1* mRNA was significantly lower in the *FBN1* het KO cells than in WT cells (Fig. 1b). On the other hand, no significant difference was observed in the amount of *FBN1* preRNA before splicing between *FBN1* het KO and WT cells, which was as expected from the results of homozygous KO pigs (Supplementary Fig. 1a).

Previous studies have reported that differences in primary cultured cell passage number can affect gene expression^18–20^. In addition, disease onset tends to vary among Marfan’s patients even in the same pedigree. To investigate the regulation of *FBN1* expression in the *in vitro* het KO cell cultures, we evaluated on the passage number of *FBN1*-expressing fibroblast cells by RT-PCR. Differences in *FBN1* het KO cell passage number revealed that the average amounts of both *FBN1* mRNA and preRNA of the six *FBN1* het KO fibroblast cell lines were comparable between P6 (early passage) and P20 (late passage) (Fig. 1c). However, the ratio of *FBN1* mRNA to preRNA (mRNA/preRNA) in each cell line tended to decrease at P20 compared with P6. In addition, the *FBN1* mRNA level in each het KO line changed with the passage number, and expression patterns in P6 to P20 differed depending on cell line (Fig. 1d). The *FBN1* mRNA was only detected from the WT allele by RT-PCR (Supplementary Fig. 1), suggesting that transcription levels of the WT allele changed with the passage number, resulting in variable mature mRNA levels in *FBN1*-expressing fibroblast cell populations.

### Allele-distinguished DNA methylation analysis within the *FBN1* CpG island shore in *FBN1* het KO fibroblast cells

**Figure 2:**
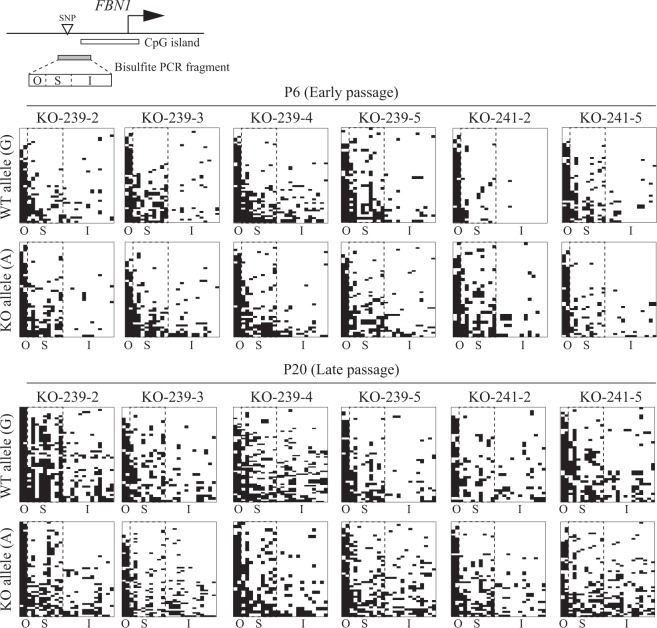
DNA methylation analysis of *FBN1* CpG island shores of WT and KO alleles in *FBN1* het KO fibroblast cells. A diagram of the porcine *FBN1* upstream region is illustrated in the upper panel. The SNP between the WT and KO alleles identified in this study is located in the *FBN1* CpG island shore. Sequenced sodium bisulfite PCR fragments including the *FBN1* CpG island shore were divided into three sub-regions based on methylation status (O, outside the *FBN1* CpG island shore. S, shore region. I, CpG island.). The DNA methylation status of each allele in *FBN1* het KO fibroblast cells is represented in the lower panel. Depending on the SNP (G or A) present in the sodium bisulfite PCR fragments, sequenced reads were defined as WT (G) or KO (A) alleles for each cell line collected at P6 (early passage) and P20 (late passage). Horizontal and vertical axes indicate the position of the CpG sites (O, 2 CpGs; S, 9 CpGs; I, 13 CpGs) and the number of sequenced reads (29 to 45 reads), respectively. White and black bars indicate unmethylated and methylated CpGs, respectively, consistent with our previous study^15^. CpGs within the two dotted lines are in the shore region (S) and whose RM allele ratio was calculated in the present study.

To investigate the changes of *FBN1* mRNA expression with passage number in *FBN1* het KO cells, we first focused on DNA methylation status within the *FBN1* CpG island shore of each allele with sodium bisulfite sequencing (Fig. 2). The SNP that we found in the CpG island shore was a C/T SNP difference on one strand between the WT and KO alleles. However, bisulfite modification eliminates the information of the C/T SNP because unmethylated cytosines are converted to uracil and then thymine during PCR amplification. Thus, allele-distinguishable bisulfite sequencing was performed using a primer set that could amplify the opposite strand containing G/A SNPs to distinguish the WT (G) and KO (A) alleles (Fig. 1a). Bisulfite PCR uses only for one strand of the double-stranded genomic DNA; the two strands are initially complementary to each other but become non-complementary with bisulfite treatment. Based on the SNPs present on the opposite strand, the G/A SNP is not affected by bisulfite treatment and retains the SNP for allele identification. We used the SNP within the *FBN1* CpG island shore (i.e. G: WT allele, A: KO allele) to analyze the DNA methylation status of six *FBN1* het KO cell lines at P6 and P20. Based on the methylation level, we previously divided the *FBN1* CpG island shore into three sub-regions: the constitutively hypermethylated outside region (O), the constitutively hypomethylated CpG island region (I), and the intermediately and differentially methylated core region of the CpG island shore (S) between O and I^17^. Focusing on the hypomethylated allele ratio rather than a conventional DNA methylation rate of the examined region, the proportion of a certain cell type can be more precisely estimated in heterogeneous cell populations such as tissues^15^. In the present study, either hypermethylated or hypomethylated patterns were observed in the S region of the sequenced fragments in *FBN1* het KO cells (Fig. 2). Thus, we calculated the RM allele ratio of the WT and KO alleles based on the methylation information of nine CpG sites contained in the S region that is strongly associated with *FBN1* expression (Supplementary Fig. 2b), and a fragment with seven or more unmethylated CpGs was defined as an RM allele in accordance with the criteria used in our previous study^15^. Furthermore, *FBN1* het KO cells possess two *FBN1* alleles, of which one is normal (WT) and the other is mutated (KO) allele. In other words, one *FBN1* het KO cell contains one WT allele, whose hypomethylation results in the expression of functional *FBN1* mRNA. Thus, under the experimental condition that bisulfite PCR bears no PCR bias as confirmed in our previous study^15^, it should be emphasized here that the calculation of the RM allele ratio that focuses only on the WT allele is equivalent to the estimation of the proportion of the cells that contain the hypomethylated and *FBN1*-producing WT allele in the cultured *FBN1* het KO cell population. Taking advantage of this correspondence, we determined the ratio of the cells that were hypomethylated in the CpG island shore of the WT allele, which likely resulted in sufficient expression of functional WT mRNA in the primary fibroblast cell lines.

**Figure 3:**
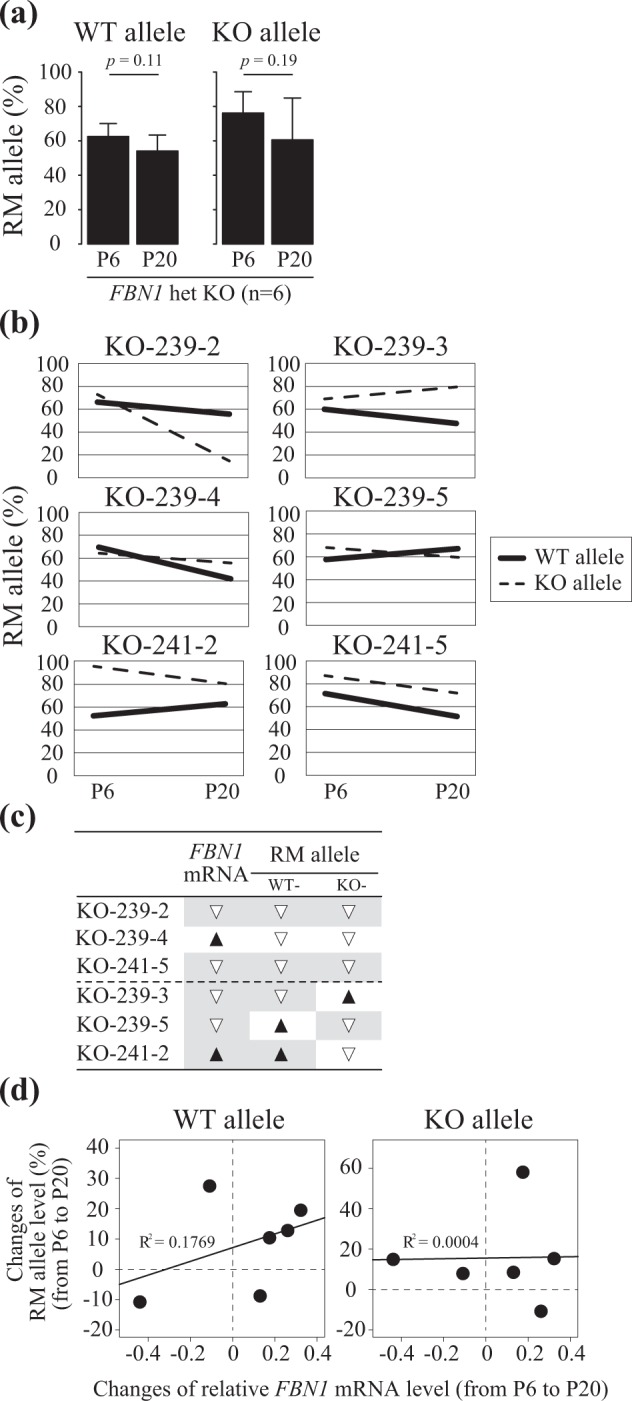
Stochastic DNA methylation changes on both the WT and KO alleles after passage of *FBN1* het KO fibroblast cells. (**a**) RM allele ratio of the *FBN1* het KO fibroblast cells (n = 6) at P6 and P20. In the S region, sequenced reads with ≥75% unmethylated CpG sites (i.e. ≥7 out of nine CpGs) were defined as RM alleles. Based on the sodium bisulfite sequencing data in Fig. 2, RM allele ratios were calculated for the WT and KO alleles, respectively, and are shown as average ± SD. Statistical comparisons of the RM allele ratios were performed using the Student’s *t*-test, and statistical significance was set as *p* < 0.05. (**b**) Changes in RM allele ratios of *FBN1* het KO fibroblast cells from P6 to P20. Solid and dotted lines indicate the RM allele ratio of WT and KO alleles, respectively. (**c**) Summary of changes in RM allele ratios and *FBN1* mRNA levels in *FBN1* het KO fibroblast cells. The data of the *FBN1* mRNA is presented in Fig. 1. Black and white triangles indicate the increase and decrease in RM allele ratios and *FBN1* mRNA levels from P6 to P20, respectively. (**d**) Correlation between *FBN1* mRNA level and RM allele ratio. The difference in *FBN1* mRNA level and RM allele ratio between P6 and P20. Closed circles represent the data of each *FBN1* het KO fibroblast cell (n = 6).

Based on the sodium bisulfite sequencing data (Fig. 2), the RM allele ratio of six *FBN1* het KO cell lines generally decreased in P20 compared with P6 in both WT and KO alleles (Fig. 3a), suggesting that changes in DNA methylation status occurred during cell proliferation and passage. In addition, RM allele ratios of both WT and KO alleles decreased from P6 to P20 in three *FBN1* KO cell lines (KO-239-2, KO-239-4, and KO-241-5; Fig. 3b). This decrease in the RM allele ratio indicated *de novo* DNA methylation resulting in DNA hypermethylation within the CpG island shore region during *in vitro* culture of the fibroblast cells. In particular, in the KO-239-2 line, the RM allele ratio of the KO allele dramatically decreased from P6 to P20 compared with that of the WT allele. On the other hand, RM allele ratios of the WT and KO alleles changed differently in the other three cell lines (KO-239-3, KO-239-5, and KO-241-2) (Fig. 3b). To exclude the possibility that the single nucleotide difference at the SNP site affected DNA methylation, the same analysis was performed using littermate WT pig-derived fibroblast cells and showed that the direction of the changes in the RM allele ratio from P6 to P20 were identical between the two WT alleles (Supplementary Fig. 3). Among the *FBN1* het KO cell lines, the variance in the RM allele ratio of the WT and KO alleles were larger in the KO allele than the WT allele (Supplementary Fig. 4), suggesting that in the *FBN1* het KO cells the regulation of *FBN1* expression by DNA methylation differed between the WT and KO alleles. Overall, four out of the six *FBN1* het KO cell lines exhibited the same changes in *FBN1* mRNA level and RM allele ratio between P6 and P20 based on the SNP in the WT allele (Fig. 3c). Considering that each het KO cell has one WT allele, the decrease in WT RM allele ratios in these four het KO cell lines likely indicate that the proportion of the cells that can transcribe functional *FBN1* mRNA have decreased over time. By plotting the changes in *FBN1* mRNA and the RM allele ratio from P6 to P20 for each cell line, a higher correlation was observed for the WT allele than the KO allele, which was expected from their WT RM allele ratios (Fig. 3d). Since the KO allele-derived transcripts are degraded after splicing (Supplementary Fig. 1) and *FBN1* expression is regulated by DNA methylation (Supplementary Fig. 2), the functional *FBN1* mRNA levels are controlled by DNA methylation within the CpG island shore of the WT allele alone in *FBN1* het KO fibroblast cells. Furthermore, *FBN1* het KO fibroblast cells (KO-239-2 and KO-239-4) were easier to detach from culture dishes with trypsin/EDTA treatment *in vitro* than WT fibroblast cells (WT-239-1) (Supplementary Fig. 5a), and the amount of *FBN1* mRNA in detached het KO fibroblast cells was significantly lower than that in WT cultures (Supplementary Fig. 5b). Since the microfibril protein, fibrillin, has an important role in the formation of extracellular matrix and cell adhesion^21,22^, a varied adhesion phenotype among fibroblast cell lines reflects the amount of functional *FBN1* mRNA transcribed from the WT allele.

### Regulation of *FBN1* mRNA by DNA methylation of the WT allele in ear cartilage of *FBN1* het KO pigs

**Figure 4:**
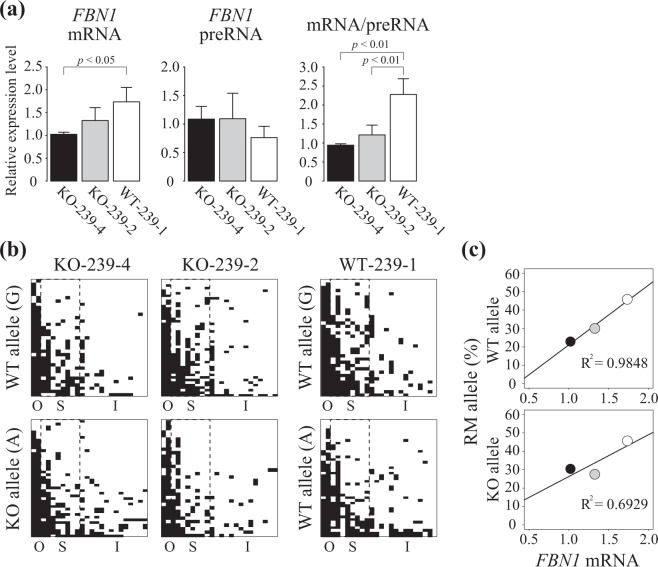
Allele-distinguished DNA methylation analysis of ear cartilage of the *FBN1* het KO pigs. (**a**) Expression levels of the *FBN1* mRNA and preRNA in the ear cartilage of *FBN1* het KO (KO-239-4, KO-239-2) and WT (WT-239-1) pigs was measured by RT-PCR. The relative expression levels were normalized to *GAPDH* expression. The mRNA/preRNA data indicate the ratio of *FBN1* mRNA in all transcribed *FBN1* RNA. The expression levels are shown as mean ± SD (n = 3). Statistical comparisons of the expression levels were performed using the Student’s *t*-test, and statistical significance was set as *p* < 0.05. (**b**) Allele-distinguished DNA methylation analysis of the same ear cartilage samples from *FBN1* het KO and WT pigs was conducted by sodium bisulfite sequencing. Depending on the SNP in the *FBN1* CpG island shore, the WT (G) or KO (A) alleles present in the *FBN1* het KO pigs were determined in each sequenced read. Although the WT pig analyzed has two WT alleles, one is derived from a WT pig with two G alleles and the other is derived from a WT allele from the *FBN1* het KO pig (A). O: hypermethylated outside region. S: variably methylated shore region. I: hypomethylated CpG island. (**c**) Correlation between *FBN1* mRNA level and the RM allele ratio of the S region. Black, gray, and white circles indicate ear-cartilage data from KO-239-4, KO-239-2, and WT-239-1, respectively. In the WT-239-1, the RM allele ratio was calculated using all the sequenced clones of both WT-derived and het-KO-derived *FBN1* CpG island shores and was used in both WT- and KO-allele plots without distinguishing the two alleles because two WT alleles were not determined for the WT-239-1 pig in the RT-PCR analysis.

Next, we attempted to analyze whether *FBN1* mRNA transcription is regulated by DNA methylation of the WT allele *in vivo* using sodium bisulfite sequencing and RT-PCR of porcine tissue to distinguish the WT and KO allele SNPs within the *FBN1* CpG island shore and the 1-bp deletion site in *FBN1* transcript. It has been reported that *FBN1* is highly expressed in a cartilage tissue^21^ and our data also indicated that *FBN1* mRNA was clearly detected in ear cartilage of *FBN1* het KO pigs, KO-239-4 and KO-239-2 (Fig. 4a). Differences in the *FBN1* expression level in ear cartilage was observed between the two *FBN1* het KO pigs analyzed and the *FBN1* mRNA level of KO-239-4 tended to be lower than that of KO-239-2. This lower *FBN1* mRNA level was also observed in fibroblast cells derived from the same pigs (data not shown). In addition, although the *FBN1* preRNA level in ear cartilage was similar between the two *FBN1* het KO pigs, a lower mRNA to preRNA ratio was observed in KO-239-4 than in KO-239-2 (Fig. 4a). Analysis of the allele-dependent DNA methylation status of the *FBN1* CpG island shore (Fig. 4b) and the *FBN1* mRNA level in ear cartilage revealed a higher correlation in the WT allele than in the KO allele (Fig. 4c). These results indicate that functional *FBN1* mRNA levels are controlled by DNA methylation on the WT allele in *FBN1*-expressing tissues of the het KO pigs, and is probably a variable outcome of changes in DNA methylation during *in vivo* ontogenetic growth and/or aging. Similarly, the ratio of cells representing the DNA hypomethylated state in the CpG island shore of the WT allele is most likely reflected in the total amount of the functional *FBN1* mRNA in the tissue.

### DNA methylation status in the upstream region of *FBN1* in human somatic cells

**Figure 5:**
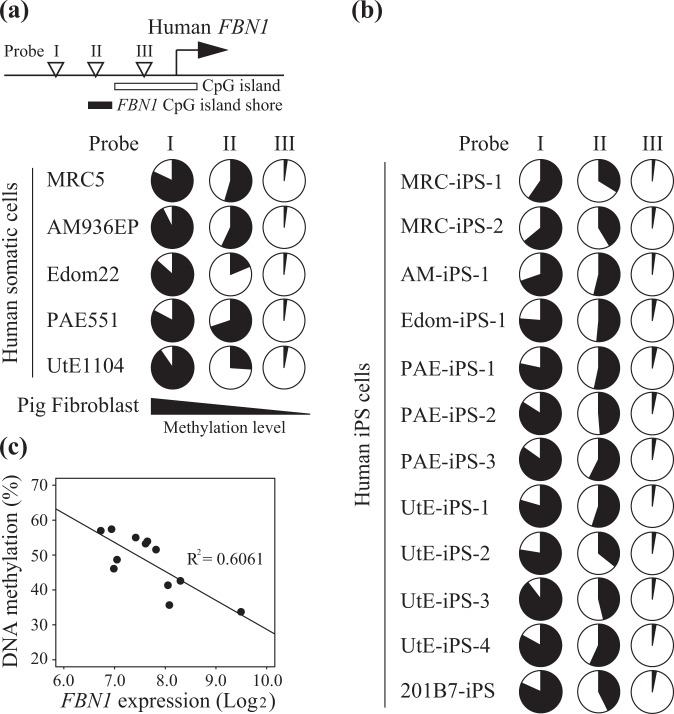
DNA methylation status of *FBN1* CpG island shore and *FBN1* expression in human cells. The DNA methylation status of five human somatic cell lines (MRC5, AM936EP, Edom22, PAE551, and UtE1104) (**a**) and twelve human iPS cell lines (**b**) were analyzed with the Infinium Human Methylation450K BeadChip. In the human *FBN1* upstream region, three probes (probe I, II, and III) that are located in the outside region (probe I), the CpG island shore (probe II), and inside the CpG island (probe III) of the human *FBN1* locus. DNA methylation levels are presented as a pie chart; black and white areas indicate the percentages of methylated and unmethylated CpGs, respectively. (**c**) The correlation between *FBN1* mRNA level and DNA methylation level of *FBN1* CpG island shore (probe II) was calculated for human iPS cell lines (n = 12). *FBN1* expression and DNA methylation data were based on the gene expression array and the Infinium Human Methylation450K BeadChip, respectively.

We previously reported that the *FBN1* CpG island shore is highly conserved in pig and human genomes^17^. To investigate whether epigenetic regulation of *FBN1* observed in porcine fibroblast cells is conserved in human cells, we analyzed the DNA methylation status of the upstream region of *FBN1* in five human somatic cell lines (MRC5, AM936EP, Edom22, PAE551, and Ute1104). In the Infinium Human Methylation450K BeadChip, three probes (probe I, II, and III) were located upstream of the human *FBN1*; probe I and III were hyper- and hypo-methylated, respectively, in all five human cell lines (Fig. 5a). However, probe II is located within the *FBN1* CpG island shore of the human *FBN1* and had variable and moderate DNA methylation levels (18.8 to 69.9%) that depended on the cell line.

Next, the correlation between DNA methylation and *FBN1* expression in human somatic cells was analyzed. However, the human somatic cells examined exhibited high levels of *FBN1* expression; therefore, these data were not suitable for verifying the correlation between DNA methylation and *FBN1* expression. In our previous study, human induced pluripotent stem (iPS) cells were shown to differ greatly in DNA methylation status depending on the cell line^23^. Because of this, further analysis was performed using human iPS cells. DNA methylation analysis using the Infinium Human Methylation450K BeadChip revealed that human iPS cells also exhibited moderate DNA methylation levels of probe II, which is located in the *FBN1* CpG island shore. As expected, these methylation levels varied based on the cell lines examined (Fig. 5b). For the human iPS cells, the DNA methylation level of the *FBN1* CpG island shore and the *FBN1* mRNA level (analyzed using a gene expression array) showed a significantly negative correlation (Fig. 5c). In the porcine samples (Figs. 3d and 4c), a positive correlation was observed when plotting the percentage of alleles where the gene was activated (RM allele) and the level of *FBN1* mRNA expression. Conversely, in human iPS cells (Fig. 5c) a negative correlation was observed between the DNA methylation level (an indicator of cell ratio when the gene is in a suppressed state) and *FBN1* mRNA expression. Therefore, both in pigs and humans, DNA methylation in the CpG island shore plays a role in the suppression of *FBN1* mRNA expression. These results are consistent with data of porcine fibroblast cells and suggest that the human *FBN1* is also likely to be regulated by DNA methylation of the *FBN1* CpG island shore.

## Discussion

In the present study, we investigated how DNA methylation status within the *FBN1* CpG island shore of the WT and KO alleles regulates *FBN1* expression using allele-specific SNPs in *FBN1* het KO fibroblast cells as the tool. Expression levels of *FBN1* mRNA differed among fibroblast cell lines and in ear cartilage from littermate het KO pigs. These differences in *FBN1* mRNA levels were also observed in human Marfan patients^5^. In addition, RM allele ratios differed among *FBN1* het KO samples, with the WT allele exhibiting a higher correlation with *FBN1* mRNA level than the KO allele. A combination of varied DNA methylation of the WT-allele and degradation of KO-allele-derived preRNA suggests that the functional *FBN1* mRNA level in cell populations or tissues of het KO pigs is mainly regulated by DNA methylation of the WT allele. Thus, it is becoming more important to investigate epigenetic regulation of WT alleles in Marfan patients with null KO allele and its animal model of our *FBN1* het KO pigs.

**Figure 6:**
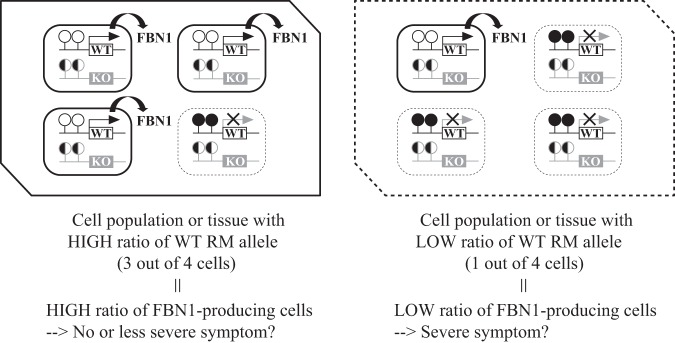
A model for *FBN1* gene regulation by varied DNA methylation of the CpG island shore of *FBN1* het KO cells/tissues. In the *FBN1* het KO cells and tissues, the amount of functional *FBN1* mRNA is regulated by DNA methylation status within the *FBN1* CpG island shore of the WT allele. The null KO allele does not contribute any FBN1 function because the *FBN1* preRNA includes a premature stop codon and degraded after splicing coupled with translational initiation. The WT allele is hypomethylated in het KO cells and therefore, the total amount of the functional *FBN1* mRNA is sufficient in tissue (cell population), regardless of the methylation status of the KO allele (left panel). In contrast, the WT allele is hypermethylated and functional *FBN1* mRNA is not sufficiently transcribed in the tissue or cell population (right panel). Open and closed circles represent unmethylated and methylated CpGs within the *FBN1* CpG island shore, respectively; while black and white circles indicate either methylated or unmethylated CpGs. White and gray squares represent WT and KO alleles of *FBN1*, respectively.

We produced *FBN1* het KO pigs, in which WT and KO alleles could be distinguished using a SNP in the *FBN1* CpG island shore. This allowed us to distinguish between WT and KO alleles, and calculate the RM allele ratio of the WT allele that directly represents the proportion of the cells with hypomethylated CpG island shores in the WT allele. The cells with the hypomethylated WT allele most likely express a sufficient level of WT *FBN1* mRNA in a certain cell population or tissue (Fig. 6). In contrast, the DNA methylation status (RM allele ratio) of the KO allele should not affect functional mRNA levels because a premature stop codon results in nonsense-mediated RNA decay even if transcript is produced from the KO allele. However, this relation between cell proportion and gene expression level cannot be applied to samples without allele-distinguishable SNPs in the differentially DNA-methylated CpG island shore of het KO cells. Thus, as a practical reason for analyzing epigenetic changes on the WT allele in addition to modeling haploinsufficiency diseases, our *FBN1* het KO fibroblast cells distinguishable of the WT and KO alleles are useful for understanding the molecular mechanisms of stochastic downregulation of the responsible gene in relation to epigenetic ambiguity.

Our previous study showed that the upstream region of the porcine *FBN1*, especially the CpG island shore, is highly homologous to the human *FBN1*, including the number of CpGs^17^. In the present study, we demonstrated variable DNA methylation levels within the *FBN1* CpG island shore in several human cell lines. In addition, a significant correlation between DNA methylation and *FBN1* expression was observed among human iPS cell lines. This suggests that human *FBN1* is also likely to be regulated by DNA methylation within the *FBN1* CpG island shore. Our findings on porcine *FBN1* CpG island shore suggest a causal relationship between the expression level of *FBN1* mRNA and the RM allele ratio of the WT allele in pigs. Therefore, this model can be applied to the characterization of pathogenic molecular mechanisms of Marfan syndrome in humans.

One cause of Marfan syndrome is haploinsufficiency, and Marfan patients with a low level of the *FBN1* expression from a single WT allele have an increased risk of presenting several Marfan symptoms^5^. In addition, disease onset and severity differ among affected individuals with similar genetic abnormalities. Taken together with the results of this study, disease onset and severity of haploinsufficient Marfan syndrome involves DNA hypermethylation on the WT allele within the *FBN1* CpG island shore that results in decreased functional *FBN1* mRNA levels. Throughout development, the DNA methylation profile changes along with patterns of gene expression in developmental- and cell type-specific manners^12,13^. Abnormalities in epigenetics, including DNA methylation, are known to be involved in the onset of various diseases, such as cancer^24^. Thus, when accompanied by genetic mutation, stochastic epigenetic errors in haploinsufficient genes themselves during development and after birth are important factors that affect disease onset in heterozygous individuals.

Low levels of the *FBN1* mRNA were observed *in vivo* in ear cartilage tissue from het KO pigs and fibroblast cells. In addition, *FBN1* mRNA level was strongly correlated with RM allele ratios of the WT allele in ear cartilage samples, in consistent with *in vitro* fibroblast data. The SNP-containing het KO pigs we analyzed in the present study did not exhibit typical symptoms of Marfan syndrome during the experiment, and the decrease in mRNA levels were relatively mild. Furthermore, although differences in the expression levels of *FBN1*-related genes, such as *TGFb* and *MMP2*, were observed in our *FBN1* het KO samples, these discrepancies were not identical to those observed in Marfan syndrome. This was likely due to the lack of overt phenotypes in the *FBN1* het KO pigs analyzed. Autosomal recessive diseases can be simply reproduced in animal models by producing homozygotes of null alleles, whereas a difficulty in studying haploinsufficient autosomal dominant diseases is their variable penetrance and expressivity as is also the case for Marfan syndrome and its animal models of our *FBN1* het KO pigs. Thus, one of the next issues to be addressed is whether SNP-containing *FBN1* het KO pigs with apparent Marfan symptoms have both decreased *FBN1* mRNA levels and RM allele ratios. The present study indicates that DNA hypermethylation of the *FBN1* CpG island shore led to decreased mRNA levels, suggesting that artificial induction of DNA hypermethylation accelerates disease onset and manifestation of symptoms. Although epigenetics-modifying chemicals have mainly been studied for gene activation by targeting tumor suppressor genes for cancer therapy, the methyl-group donor, S-adenosyl methionine (SAM), or folate are possible candidates for the induction of DNA hypermethylation^25^. Thus, an alternative approach to investigating induced DNA hypermethylation of the *FBN1* CpG island shore, which likely leads to efficient production of haploinsufficient disease models, is to feed *FBN1* het KO pigs a high folate- or SAM-containing diet and see whether disease phenotypes manifest.

Our previous study on the epigenetic regulation of porcine preimplantation stage embryos suggested that DNA methylation of *FBN1* is erased until the blastocyst stage and re-methylation occurs later during embryonic development^17^. In the present study, we found that the DNA methylation level of the *FBN1* CpG island shore changed during the cell culture passages. These results suggest that DNA methylation status of the *FBN1* CpG island shore dynamically changes depending on the developmental stage and cellular environment, and formation of DNA methylation patterns is crucial for the *FBN1* expression in cells/tissues. In addition, the CpG island shore is located at the boundary of a constitutively unmethylated CpG island and constitutively methylated outside region, and this locational feature of the CpG island shore confers a variable outcome of random hypo- or hyper-methylation in addition to tissue/cell-type specific regulation. In the porcine and human *FBN1* genes, the CpG island shore is located in close proximity to the transcriptional start site in the promoter region that is regulated by DNA methylation. Thus, *FBN1*-haploinsufficient Marfan syndrome including our Marfan model pigs may present early onset when the methylation pattern of the *FBN1* CpG island shore of the WT allele is accidentally disturbed after birth to a DNA hypermethylation direction, resulting in decreased functional mRNA/protein levels in connective tissues. On the other hand, if such disturbances in DNA methylation patterns in the *FBN1* CpG island shore occurs at very low levels in each cell or in very few cells in tissues, it is possible that Marfan syndrome disease onset could be delayed or not occur at all. Moreover regarding *FBN1*-dominant-negative Marfan syndrome, our finding that allele-dependent DNA methylation correlated with mRNA expression suggests that DNA hypomethylation of the mutant dominant-negative allele could exaggerate Marfan symptoms. Although any factors causing this type of ambiguity in *FBN1* CpG island shore DNA methylation patterns are still not determined, ambiguous epigenetic errors within the *FBN1* CpG island shore are a new insight into elucidating haploinsufficient Marfan syndrome onset with better accuracy.

In conclusion, DNA methylation of the *FBN1* CpG island shore on the WT allele regulates functional *FBN1* mRNA levels in *FBN1* het KO cells/tissues. At present, hundreds of haploinsufficient diseases are known, but the mechanism(s) underlying regulation of the responsible genes leading to disease onset remain to be elucidated. Null mutations, in general, result in autosomal recessive genetic disease; however, the same type of null mutations in certain genes cause haploinsufficient dominant diseases, as is the case for Marfan syndrome. Induced DNA hypermethylation or epigenetic fluctuation of WT alleles as a novel modifier is likely a molecular contributor to haploinsufficiency that can further result in a wide variety of penetrance and expressivity of symptoms. About half of mammalian genes contain CpG islands in their promoter regions, and the expression of these genes can be regulated by stochastic changes of DNA methylation patterns within these CpG island shores. Therefore, it is important to characterize epigenetic fluctuations in the WT allele, together with conventional genetic null mutations in the KO allele, as part of the molecular pathogenesis of haploinsufficiency.

## Materials and Methods

### Animal care

All animal experiments were approved by the Institutional Animal Care and Use Committee of Meiji University (IACUC12-0008 and IACUC15-0001). All animal care and experimental procedures were performed in accordance with the Japan Act on Welfare and Management of Animals and all applicable regulations. The pigs were housed in a temperature-controlled room, had free access to water, and were provided with commercial feed appropriate for their growth stage (Nosan Co., Yokohama, Japan). For the autopsies, the pigs received an intramuscular injection of 1% mafoprazine mesylate (0.5 mg/kg, DS Pharma Animal Health Co., Ltd., Osaka, Japan) and midazolam (0.1 mg/kg, Takeda Pharmaceutical Co., Ltd., Tokyo, Japan), followed by an intravenous injection of sodium thiopental (Nipro ES Pharma Co., Ltd., Osaka, Japan). Anesthesia was maintained via the inhalation of isoflurane (DS Pharma Animal Health Co., Ltd.) while the pigs were exsanguinated.

### Identification of a single nucleotide polymorphism (SNP) within the porcine *FBN1* CpG island shore

In order to perform DNA methylation analysis of the *FBN1* upstream region to distinguish the normal (WT) and *FBN1*-mutated null (KO) alleles, we attempted to find single nucleotide polymorphisms (SNPs) within the *FBN1* CpG island shore by sequencing analysis. Briefly, genomic DNA was extracted from porcine peripheral blood using the NucleoSpin Blood kit (TaKaRa, Kyoto, Japan) according to the manufacture’s protocol. Purified genomic DNA was digested with *Hind*III (TaKaRa) and gel purified with a QIAquick Gel Extraction Kit (Qiagen, Hilden, Germany). Digested DNA was amplified with AccuPrime Taq DNA polymerase (Thermo Fisher Scientific, Carlsbad, CA) using specific primers for the porcine *FBN1* CpG island shore region (*FBN1*_4F: 5′-GAGTTGAGGCAATGGGAAGA-3′, *FBN1*_4R: 5′-TGTGTCTGGGAGGCACAGT-3′). PCR was performed under the following conditions: 94 °C for 2 min; 35 cycles of 94 °C for 30 s, 60 °C for 30 s, and 68 °C for 1 min. and an adenine-tailing using Taq DNA polymerase at 72 °C for 10 min. Genomic PCR products were subcloned into the pGEM-T Easy vector (Promega, Madison, WI, USA), and sequencing was performed by Applied Biosystems 3130xL (Thermo Fisher Scientific).

### Porcine *FBN1* heterozygous knockout (het KO) fibroblast cell culture

Establishment of the *FBN1* het KO fibroblast cell line was performed using porcine neonatal tail cartilage. Primary culture of porcine fibroblasts was performed as previously described^26^. Tail cartilage tissue was chopped into small pieces and placed in a type I collagen-coated dish containing minimum essential medium eagle (MEM) alpha modifications (Thermo Fisher Scientific) supplemented with 15% fetal bovine serum (HyClone, Thermo Fisher Scientific) and Penicillin-Streptomycin (Thermo Fisher Scientific). Fibroblast cells were grown in a humidified atmosphere with 5% CO_2_ at 37 °C, and sub-confluent cells were passaged and collected at the 6th and 20th passages (P6 and P20). Collected cells were stored at −80 °C until genomic DNA or RNA extraction.

### Genomic DNA extraction and sodium bisulfite reaction

Genomic DNA extraction and bisulfite conversion were carried out as described previously^14^. Genomic DNA was purified from porcine fibroblast cells and ear cartilage using the DNeasy Blood & Tissue Kit (Qiagen) according to the manufacturer’s protocol. The genomic DNA was digested with *Hind*III (TaKaRa) and gel purified with a QIAquick Gel Extraction Kit (Qiagen), and bisulfite reactions were performed with the EZ DNA Methylation-Gold Kit (Zymo Research, Irvine, CA). Bisulfite-treated DNA was amplified with BioTaq HS DNA polymerase (BIOLINE, London, UK) using specific primers for the porcine *FBN1* CpG island shore (*FBN1*_Bis_F: 5′-AGTTTTAATGTGAGTTGGATAAAAGGA-3′, *FBN1*_Bis_R: 5′-AAAAACTATACCACCTACACCAAAAA-3′). PCR was performed under the following conditions: 95 °C for 10 min; 40 cycles of 95 °C for 30 s, 60 °C for 30 s, and 72 °C for 1 min; final extension 72 °C for 2 min. The PCR products were subcloned into pGEM-T Easy vector (Promega) for sodium bisulfite sequencing.

### Gene expression analysis

Total RNA from porcine fibroblast cells and ear cartilage were extracted using the RNeasy Plus Mini and RNeasy Fibrous Tissue kits (Qiagen), respectively. First-strand cDNA synthesis was performed using a Superscript III First-strand Synthesis System (Thermo Fisher Scientific) with transcript-specific primers: *FBN1*_del1 (for *FBN1* mRNA): 5′-GCACTCTCCACGGAGGTCC-3′, *FBN1*-del3 (for *FBN1* preRNA): 5′-GCAATAGGCAAATTGAGAAACTTC-3′, and *GAPDH*: 5′-TCTGGGATGGAAACTGGAAG-3′. RT-PCR was performed using TaKaRa LA Taq (TaKaRa) with specific primers for *FBN1* mRNA (*FBN1*_RT_F1: 5′-ATGTCCCTATGGTAGTTCCTGAGA-3′, *FBN1*_RT_R1: 5′-AGTTGGAATCCTTTGTTGCACT-3′), *FBN1* preRNA (*FBN1*_RT_F3: 5′-CAGTCCCTCGACCACCAG-3′, *FBN1*_RT_R3: 5′-CCAACTTCATGACTTAAGCAGCAT-3′), and *GAPDH* (*GAPDH*_RT_F: 5′-ACCACAGTCCATGCCATCAC-3′, *GAPDH*_RT_R: 5′-TCCACCACCCTGTTGCTGTA-3′). RT-PCR was performed using the following conditions for fibroblast cells: 95 °C for 5 min; 26 cycles (*FBN1* mRNA), 33 cycles (*FBN1* preRNA), or 23 cycles (*GAPDH*) of 95 °C for 30 s, 60 °C for 30 s, and 72 °C for 1 min; final extension 72 °C for 10 min. For ear cartilage samples, PCR were performed with 28 cycles (*FBN1* mRNA), 33 cycles (*FBN1* preRNA), or 26 cycles (*GAPDH*).

Gene expression array data were deposited in the Gene Expression Omnibus repository (GEO; http//www.ncbi.nlm.nih.gov/geo/) with the following accession numbers: GSE20750, GSE24677, and GSE42099^23,27^. All expression data were analyzed using an Agilent-014850 Whole Human Genome Microarray 4×44 K G4112F and extracted for the specific *FBN1* gene probe (A_23_P65678). The expression data were processed by quantile normalization for analysis.

### DNA methylation analysis of the *FBN1* CpG island shore region in human somatic cells

DNA methylation data of five human somatic cell lines (MRC5, AM936EP, Edom22, PAE551, and UtE1104) and the corresponding iPS cell lines established from these somatic cells were collected from our previous study using an Infinium HumanMethylation450K BeadChip^28,29^. The data were extracted for specific probes that were located outside (probe I, cg01529092) or within (Probe II, cg14164380) the CpG island shore, as well as inside the CpG island (probe III, cg15385562). These methylation data were deposited in GEO with the following accession numbers: GSE73938, GSE99716, GSE116924, and GSE141521.

### Statistical analysis

All experiments were performed in triplicate, and the results were represented as mean values with standard deviation. All statistical analyses were performed using GraphPad Prism 8.0 software (San Diego, CA, USA). Statistical significance of two independent experiments were determined using Student’s *t*-test, and *p* < 0.05 were defined as significant.

## Supplementary information

Supplementary Information.
