# Supplementary material for: DNA methylation ambiguity in the Fibrillin-1 (FBN1) CpG island shore possibly involved in Marfan syndrome
Source: Sci Rep. 2020 Mar 24;10:5287. doi: 10.1038/s41598-020-62127-3 (PMC7093481; doi:10.1038/s41598-020-62127-3)
Supplement: Supplementary file 1 — Supplementary Information. [file 41598_2020_62127_MOESM1_ESM.pdf]

Supplementary information

DNA methylation ambiguity in the *Fibrillin-1* (*FBN1*) CpG island shore possibly involved in Marfan syndrome

Yoshikazu Arai, Kazuhiro Umeyama, Natsumi Okazaki, Kazuaki Nakano, Koichiro Nishino, Hiroshi Nagashima, and Jun Ohgane

Materials and Methods

Real-time RT-PCR

Total RNA from porcine fibroblast cells was extracted using the RNeasy Plus Mini Kit and RNeasy Fibrous Tissue Kit (Qiagen). First-strand cDNA synthesis was performed using a Superscript III First-strand Synthesis System (Thermo Fisher Scientific) with random hexamers. Real-time RT-PCR was performed using Premix Ex Taq (TaKaRa) with specific primers and TaqMan probes for *FBN1* mRNA (*FBN1*_Ex10: 5′- GACCGCAAATTCCAGTC -3′, *FBN1*_Ex11: 5′- TAATCAGTGACGTTGACAG -3′, and *FBN1*_Probe: 5′- CGACCACCAGTGGAATATCCATAT -3′) and *ACTB* (*ACTB*_F: 5′- GCCCTCCTTCTTGGGCATG -3′, *ACTB*_R: 5′- CAGCACCGTGTTGGCGTAG -3′, and *ACTB*_Probe: 5′- TCCTTCCTGATGTCCACGTCGCACTTC -3′). Data was normalized to *ACTB* expression, and gene expression levels were calculated using the $\Delta\Delta$Ct method.

Luciferase assay

The *FBN1* CpG island shore region of the porcine *FBN1* was amplified with specific primers (*FBN1*_region4_F: 5’- GAGTTGAGGCAATGGGAAGA -3’, and *FBN1*_region4_R: 5’- TGTGTCTGGGAGGCACAGT -3’) by genomic PCR. The PCR fragment was cloned into a CpG-free pCpG-L vector, which was kindly provided by Dr. Michael Rehli[1], and the methylated construct was obtained using *Sss*I methylase (New England BioLabs). Porcine fibroblasts were co-transfected with a reporter constructs and the pRL-TK vector by Neon Transfection System

(Thermo Fisher Scientific). Luciferase activities were determined using the Dual-Luciferase Reporter Assay system (Promega) 48 h after transfection. Promoter activity was normalized to Renilla luciferase activity. Independent experiments were performed three times.

Cell detachment assay

To investigate the ability of cell adhesion in *FBN1* het KO and WT fibroblast cells, we performed a cell detachment assay. The cell culture methodology is described in the Materials and Methods section of the present paper. Using *FBN1* het KO (KO-239-2 and KO-239-4) and WT (WT-239-1) fibroblast cells, 2.5×10^5 cells were cultured for 96 h before performing the cell detachment assay. Cell were washed with PBS(-) (Wako, Osaka, Japan) and then treated with 0.05% trypsin/EDTA (Wako) for 3 min. The detached cells in the supernatant were carefully collected and 0.05% trypsin/EDTA solution was added again to the culture dish to completely harvest the attached cells. The cells were counted using a Vi-CELL XR (Beckman Coulter, Pasadena, CA), and the ratio of the detached cells was calculated using the formula: detached cells (%) = number of detached cells in supernatant/total number of the cells (detached and attached cells)*100. Six independent cell detachment assays were performed for this analysis.

Figure legends

Supplemental Figure 1. Expression level of the *FBN1* mRNA and preRNA in *FBN1* homozygous KO fibroblast cells

(a) Expression levels of *FBN1* mRNA and preRNA in WT and *FBN1* homo KO fibroblast cells was measured by RT-PCR. The position of the primer sets are described in Fig. 1. Relative expression levels were normalized to *GAPDH.* The expression levels are shown as mean ± SD (n = 3). Statistical comparisons of the expression levels were performed using the Student's *t*-test, and statistical significance was set as $p < 0.05$. The gel images were cropped from different parts of the same gel (the original image is shown in the following Supplemental Figure 1a). (b) Real-time RT-PCR was performed with samples from WT and *FBN1* homo KO fibroblast cells. The amplification plots of *FBN1* mRNA (*FBN1*) and an internal control (*ACTB*) are shown in the left panel. The relative expression levels of the *FBN1* mRNA were normalized to those of *ACTB* using the $\Delta\Delta$Ct method.

Supplemental Figure 2. Influence of DNA methylation on the promoter activity of the *FBN1* CpG island shore

(a) *FBN1* mRNA expression in *FBN1* het KO fibroblast cells treated with 5-aza-2'-deoxycytidine (5-aza-dC). Inhibition of DNA methylation in *FBN1* het KO fibroblast cells (KO-239-2 and KO-239-4) was achieved through treatment with 0 or 5 μM of 5-aza-dC for four days. The relative expression of *FBN1* mRNA was analyzed by RT-PCR and normalized to *GAPDH.* Data is presented as mean ± SD (n = 3). Statistical analyses of mRNA expression were performed using the Student's *t*-test, and $p < 0.05$ was considered as statistically significant. (b) DNA methylation of the *FBN1* CpG island shore repressed gene promoter activity. A diagram of the *FBN1* upstream region

and reporter constructs are shown in the left panel. The *FBN1* CpG island shore was inserted into the pCpG-L luciferase vector. Mock indicates the absence of the *FBN1* CpG island shore. Methylated and unmethylated reflects the presence and absence of *in vitro* methylation, respectively, within the *FBN1* CpG island shore by *Sss*I. Open and closed circles indicate unmethylated and methylated CpG sites within the CpG island shore, respectively. Each reporter construct was transfected to porcine fibroblast cells and its luciferase activity was measured. The relative promoter activity was normalized to the activity of the co-transfected Renilla luciferase and are shown as mean ± SD (n = 3).

Supplemental Figure 3. Allele-distinguished DNA methylation analysis of porcine WT fibroblast cells

(a) DNA methylation analysis of the *FBN1* CpG island shore was performed in WT fibroblast cells by sodium bisulfite sequencing. Analyzed WT fibroblast cells were produced by crossing of *FBN1* het KO and WT pigs whose WT alleles include the SNP that we identified in the *FBN1* CpG island shore in order to distinguish the two WT alleles in the WT offspring. In the parental *FBN1* het KO pig, the CpG island shore contained the thymine (T) SNP with a complementary strand that was amplified by sodium bisulfite PCR as adenine (A) in both WT and KO alleles. The parental WT pig has a cytosine (C) SNP whose complementary strand includes a guanine (G) at the same position. Thus, F1 WT offspring pigs produced by the *FBN1* het KO and WT pigs possess two WT alleles from each parent. Depending on the SNP (G or A) nucleotide in the sodium bisulfite PCR

fragments, the origin of the parental WT alleles were identified in the sequenced reads at P6 (early passage) and P20 (late passage). Horizontal and vertical axes indicate the position of the CpG sites (O, 2 CpGs; S, 9 CpGs; and I, 13 CpGs) and the number of sequenced reads (36 to 59 reads), respectively. White and black bars indicate unmethylated and methylated CpGs, respectively. Areas within the two dotted squares represent the shore region (S) whose methylation levels were calculated in an allele-specific manner. (b) Changes in RM allele ratios in the S region from P6 to P20 in WT fibroblast cells. Solid and dotted lines indicate RM allele ratios of the WT alleles with G or A SNPs, respectively. Calculation of RM allele ratio is described in Fig. 2.

Supplemental Figure 4. The KO alleles exhibited larger variation in RM allele ratios than the WT allele in *FBN1* het KO fibroblast cells

Based on the RM allele ratios in the S region of *FBN1* het KO fibroblast cells (n = 6) at P6 and P20, coefficient of variation in WT- and KO RM allele ratios was calculated using the formula: the ratio of standard deviation/average of RM allele ratio.

Supplemental Figure 5. Cell adhesion is correlated with *FBN1* mRNA level in *FBN1* het KO fibroblast cells

(a) Cell adhesion was analyzed in *FBN1* het KO (KO-239-2 and KO-239-4) and WT (WT-239-1) fibroblast cells. After a 3-minute treatment with trypsin/EDTA, detached cells in supernatant was carefully collected, and then attached cells in dishes were harvested by additional and complete

treatment with trypsin/EDTA. Experiments were performed six times for each sample and the median of the percentage of detached cells per total cells cultured is presented as a boxplot. (b) Expression levels of *FBN1* mRNA in detached *FBN1* het KO and WT fibroblast cells. The relative expression levels of the *FBN1* mRNA were normalized to *GAPDH* and shown as mean ± SD (n = 6). Statistical comparisons of the expression levels were performed using the Student's *t*-test, and statistical significance was set as $p < 0.05$.

Reference

1. Klug M and Rehli M. Functional analysis of promoter CpG methylation using a CpG-free luciferase reporter vector. Epigenetics **1**, 127－130 (2006).

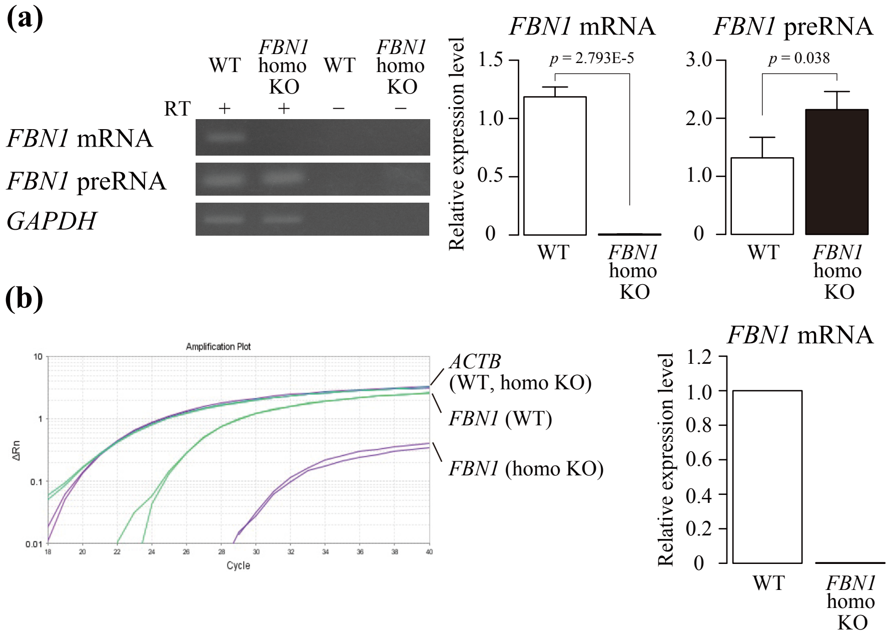

Supplemental Figure 1

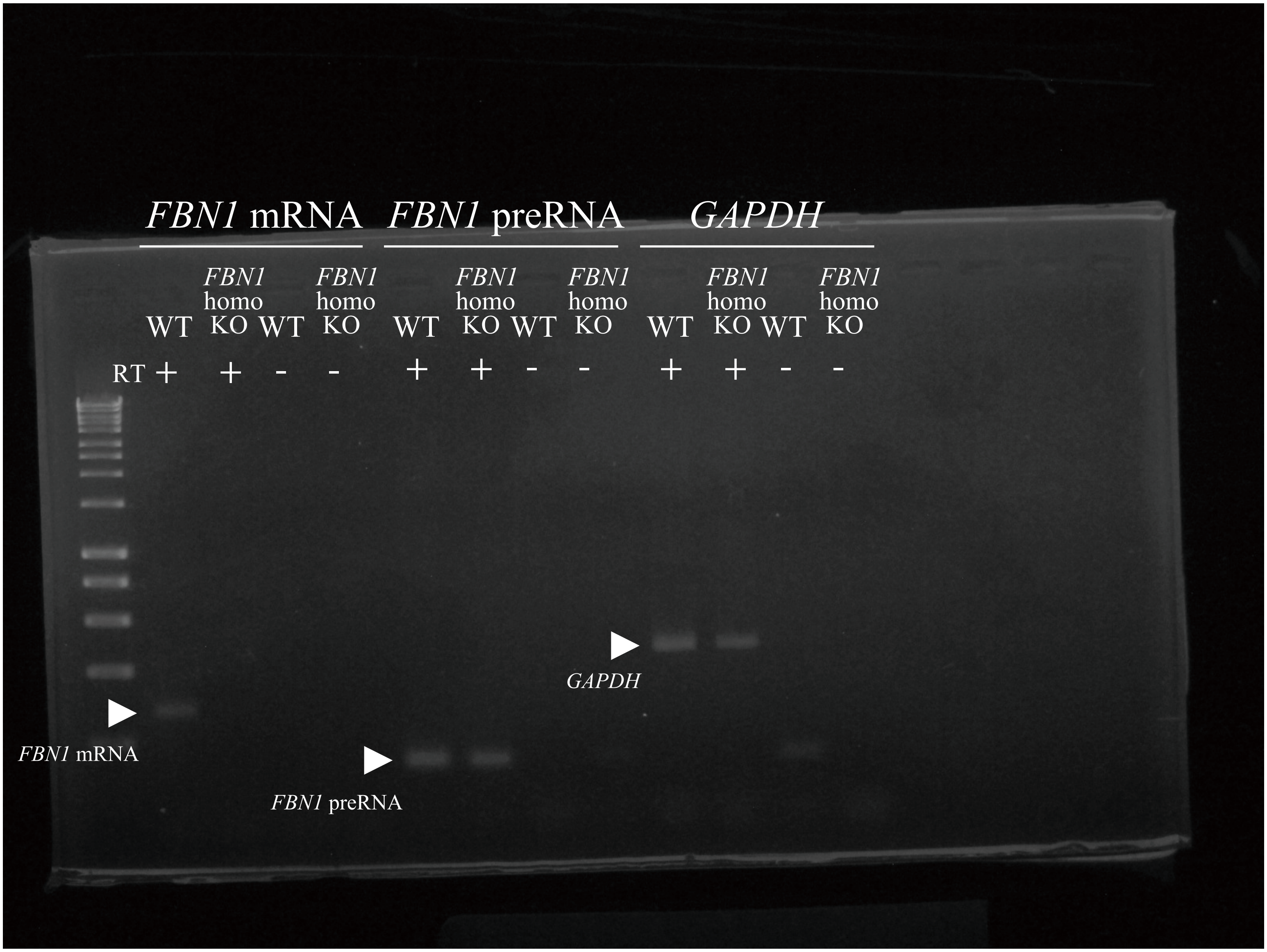

Supplemental Figure 1a

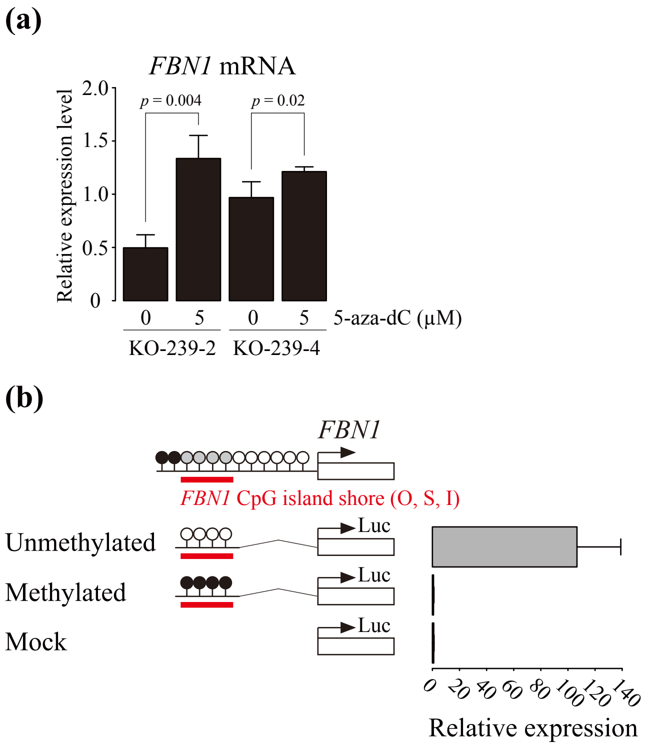

Supplemental Figure 2

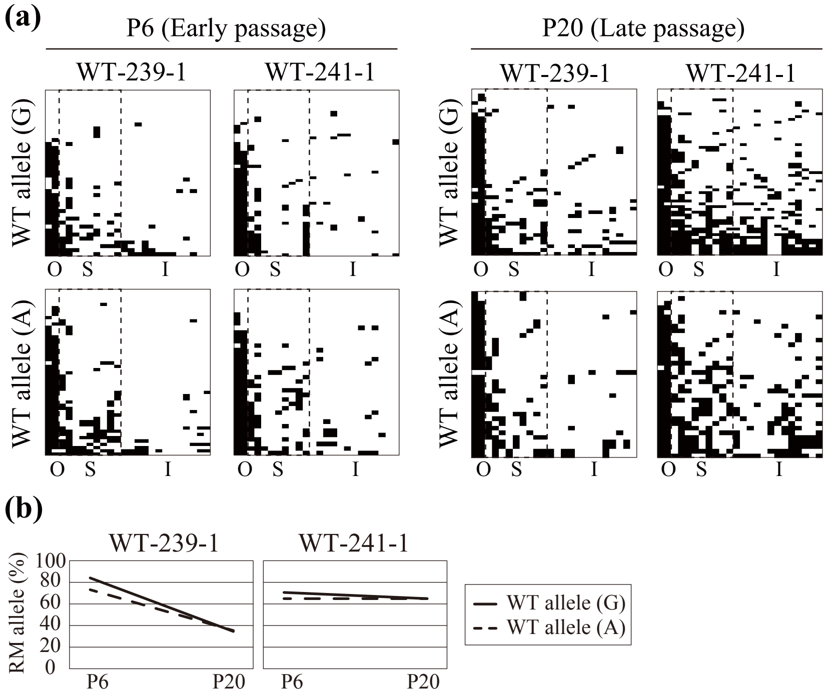

Supplemental Figure 3

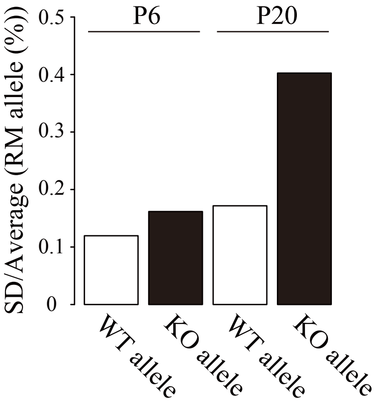

Supplemental Figure 4

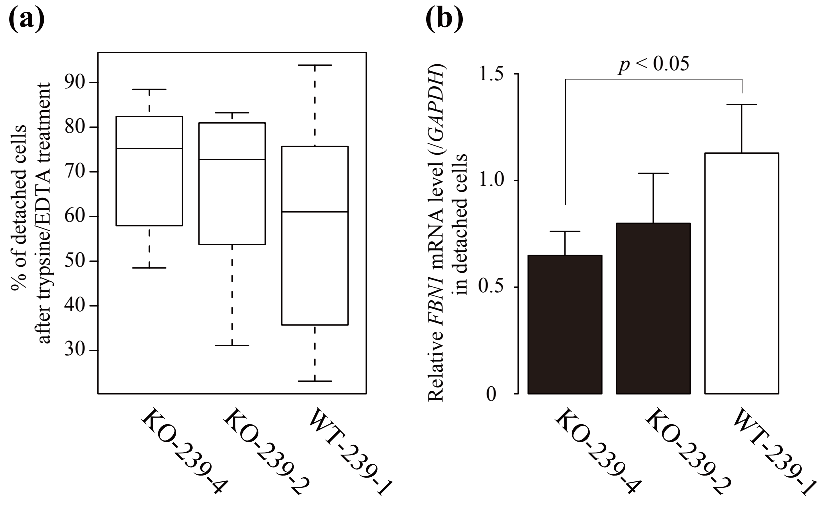

Supplemental Figure 5
